# Role of chromosomal cohesion and separation in aneuploidy and tumorigenesis

**DOI:** 10.1007/s00018-024-05122-5

**Published:** 2024-02-22

**Authors:** Debananda Pati

**Affiliations:** Texas Children’s Cancer Center, Department of Pediatrics Hematology/Oncology, Molecular and Cellular Biology, Baylor College of Medicine, 1102 Bates Avenue, Houston, TX 77030 USA

**Keywords:** Cohesin, Cancer, Chromosomal instability, Separase, Rad21, Stag2

## Abstract

Cell division is a crucial process, and one of its essential steps involves copying the genetic material, which is organized into structures called chromosomes. Before a cell can divide into two, it needs to ensure that each newly copied chromosome is paired tightly with its identical twin. This pairing is maintained by a protein complex known as cohesin, which is conserved in various organisms, from single-celled ones to humans. Cohesin essentially encircles the DNA, creating a ring-like structure to handcuff, to keep the newly synthesized sister chromosomes together in pairs. Therefore, chromosomal cohesion and separation are fundamental processes governing the attachment and segregation of sister chromatids during cell division. Metaphase-to-anaphase transition requires dissolution of cohesins by the enzyme Separase. The tight regulation of these processes is vital for safeguarding genomic stability. Dysregulation in chromosomal cohesion and separation resulting in aneuploidy, a condition characterized by an abnormal chromosome count in a cell, is strongly associated with cancer. Aneuploidy is a recurring hallmark in many cancer types, and abnormalities in chromosomal cohesion and separation have been identified as significant contributors to various cancers, such as acute myeloid leukemia, myelodysplastic syndrome, colorectal, bladder, and other solid cancers. Mutations within the cohesin complex have been associated with these cancers, as they interfere with chromosomal segregation, genome organization, and gene expression, promoting aneuploidy and contributing to the initiation of malignancy. In summary, chromosomal cohesion and separation processes play a pivotal role in preserving genomic stability, and aberrations in these mechanisms can lead to aneuploidy and cancer. Gaining a deeper understanding of the molecular intricacies of chromosomal cohesion and separation offers promising prospects for the development of innovative therapeutic approaches in the battle against cancer.

## Introduction

Cells serve as the fundamental units of our bodies, and they divide through complex processes like mitosis and meiosis. These processes involve a series of intricate steps that culminate in the replication and separation of the cell’s genetic material with very high fidelity, ultimately yielding two genetically identical daughter cells [1].

A critical aspect of mitosis is the faithful replication of the cell’s DNA during the S phase. Following this, sister chromatids, which are pairs of replicated chromosomes, are held together by a remarkable group of proteins known as the cohesin complex [2]. This complex acts as a molecular glue, maintaining the cohesion of sister chromatids until the metaphase of cell division [3–5]. During the transition from metaphase to anaphase, the replicated chromosomes are meticulously separated after the removal of the cohesin complex [6, 7]. This process ensures the precise distribution of genetic material into each daughter cell [8]. Any malfunction in this process can result in chromosomal anomalies leading to abnormal chromosome content, known as aneuploidy, and more likely to result in cell death. However, the cells that overcome the aneuploidy stress can result in uncontrolled cell growth, a defining characteristic of human tumors [9–12]. Understanding the mechanisms that govern chromosomal cohesion and separation is therefore vital for comprehending how cells preserve genomic fidelity.

## Sister chromatid cohesion and separation

### Discovery of cohesin

Historically, it was thought that DNA catenation, a process where sister chromatids are intertwined at specific sites during DNA replication, held them together, much like twisted threads, and this connection needed to be unraveled at anaphase by enzymes known as type II topoisomerases [13]. In 1987, a seminal study from the Hartwell laboratory [14] provided the first evidence against the catenation model, prompting the search for factors responsible for holding sister chromatids together. However, a pivotal discovery emerged in 1997 when two independent groups of researchers made a significant discovery using genetic studies in yeast *S. cerevisiae* [15, 16]*.* Their discovery revealed that a group of proteins known as Structural Maintenance of Chromosomes (SMC), many of which were initially identified for their roles in DNA repair, played a primary role in keeping sister chromatids together [2–4]. The complex formed by these proteins is now known as “cohesion”. When yeast cells had mutations in the genes that encode these proteins, the sister chromatids separated too soon. This discovery was a significant leap in our understanding of how mitosis, the cell division process, works.

### Composition of cohesin complex and its function

In mitotic cells, cohesin is composed of a tripartite ring containing Smc1a, Smc3, Rad21, and a peripheral subunit Scc3 in yeast that, in vertebrates, exists as two closely related isoforms: the abundant STAG2 (SA2) and the less abundant STAG1 (SA1) [2]. SMC1a and SMC3 are ABC-like ATPases [17]. The N- and C-terminus of SMC molecules fold back on themselves forming antiparallel intramolecular coiled coils. SMC1a and SMC3 form a heterodimer via the hinge domain [18–20]. The C- and N-termini of RAD21 bind proximal to the head domains of SMC1a and SMC3 heterodimer, respectively, to form a triangular ring, and SCC3 (SA1/2) binds to RAD21 and SMC3 subunits to reinforce the ring [21–23], playing a critical role in facilitating cohesin’s association with DNA [24–26]. Moreover, these STAG subunits within the cohesin complex possess the capacity to engage with RNA within the cellular nucleus [26].

**Fig. 1:**
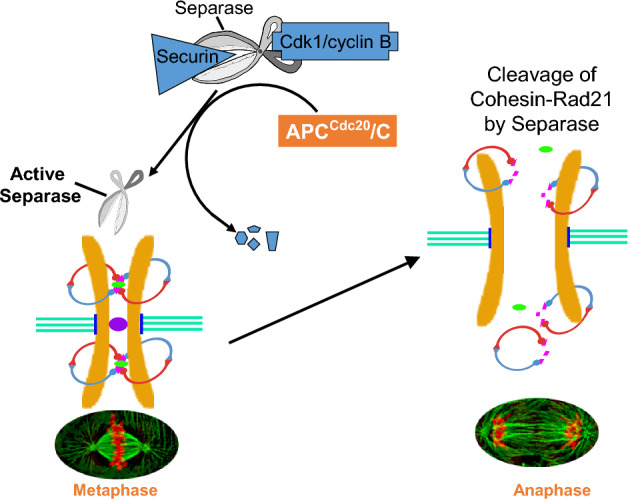
Sister chromatid cohesion and separation in human

At different stages during the cell cycle, several other proteins also dynamically associate with cohesin and regulate both cohesion and separation of sister chromatids (Table 1) [7, 8, 15, 16, 27–37]. Cohesin is loaded onto the chromatin in late telophase in vertebrates, [28, 29] which is dependent on two proteins, Scc2/Nipped-B/NIPBL and Scc4/MAU-2 [27, 31, 35]. While NIPBL/MAU-2 dimer [38] helps load cohesin onto DNA, WAPL and PDS5 help release it [39–41]. Cohesin can be loaded onto DNA at any stage of the cell cycle, but the majority of cohesin binding is temporary. To keep things stable from the S to M phase to establish cohesion between the sister chromatids, the release process (involving WAPL and PDS5) is suppressed during S phase by Sororin [36]. This suppression is facilitated through the acetylation of Smc3 at lysine residues [24, 30, 42] by acetyltransferases ESCO1 [43] and ESCO2 [44], These acetylation tags remain until the G2 and M stages, and is removed at the start of anaphase by the deacetylase Hos1/HDAC8, following RAD21 cleavage by Separase [45–47]. In early mitosis in vertebrates, during prophase, most of the cohesin from chromosomal arms is removed following phosphorylation of the cohesin component SA2 by PLK1, leaving behind a small pool of intact cohesins at the centromeres protected by the Sughosin (SGO1)-PP2A complex [37] along with some residual amounts on chromosomal arms [7, 48]. At the metaphase-to-anaphase transition, the cohesins on both centromeric and chromosomal arms are completely removed by the endopeptidase Separase, encoded by the *ESPL1* gene, which cleaves RAD21 to separate the sister chromatids [6, 8, 49, 50] (Fig. 1).

**Table 1:** Cohesin and Cohesin associated Proteins in Human

Cohesin Structural Units: RAD21, SMC1A, SMC3, SA1 (STAG1) or SA2(STAG2)	
Cohesin Associated Proteins with a role in:	
Cohesin loading	NIPBL, MAU2
Cohesion establishment	ESCO1, ESCO2
Cohesin maintenance	PDS5A, PDS5B, WAPL, CDCA5 (Sororin), SGO1, PP2A
Cohesin dissolution	ESPL1 (Separase), PLK1, CANP1 (Calpain-1), HDAC8, USP37

Cohesin is also located in centrosomes and plays a crucial role in centriole tethering. Similar to its proteolytic cleavage of RAD21 during the separation of sister chromatids in the metaphase-to-anaphase transition, Separase activity is also essential for the disengagement of centrioles [8]. Although the exact timing and the regulatory network governing centriole disengagement remain to be fully defined, the consensus is that centriole disengagement occurs during the late mitosis/early G1 phase [51–54]. This process takes place after the separation of sister chromatids, providing an additional mitotic mechanism that could impact faithful chromosome segregation. Apart from its canonical function of sister-chromatid cohesion and separation, cohesin also play roles in transcriptional regulation, DNA double-strand break repair, chromosome architecture and rearrangement, and DNA replication [23, 55, 56].

To activate transcription properly, cohesin loops chromatin, bringing distant regions together. Chromosomes contain conserved regulatory elements (CREs) with enhancers boosting transcription and insulators blocking it. Cohesins physically connect distant CREs with gene promoters in a cell-type specific manner. Depending upon cohesin’s binding to activators or insulators, it influences transcriptional outcomes either positively or negatively. Changes in cohesin levels could impact its binding to CREs and chromatin structure, altering their interaction with promoters and, consequently, gene activity. Even a slight decrease in chromatin-bound cohesin can lead to changes in the expression of many genes. However, our understanding of how cohesin is recruited and removed from transcription sites to regulate transcription is limited.

### Models of sister chromatid cohesion

Sister chromatid cohesion around the two sister chromatids is currently explained by two major models [2, 57–60]. The first model, known as the “one-ring embrace”, proposes that a single cohesin ring captures and holds together two sister chromatids throughout the cell cycle [2, 61]. Using minichromosome as a tool, Nasmyth and colleagues showed that the way cohesin and DNA connect is like a twisty knot. They did this by separating the connection between cohesin and DNA, either by cutting cohesin or cutting the DNA rings [62]. Their results supported the concept that the three parts of the Smc1–Smc3–Scc3 connection resemble more of an embrace, achieved using proteins and a cross-linker to seal them together [63]. However, later research findings challenge this idea. The alternative model, referred to as the “dimeric handcuff-model,” suggests a more complex scenario [24, 32, 57–59, 64, 65]. According to this model, multiple cohesin molecules cooperate to generate cohesion. These cohesin molecules form two separate rings, each consisting of RAD21, SMC1a, and SMC3 protein subunits. The key concept here is the formation of a handcuff-like structure, where two RAD21 molecules within these rings align in an anti-parallel orientation. The orientation is enforced by proteins STAG1 or STAG2. Mutations in the STAG-binding motif in STAG proteins reduced RAD21–RAD21 interaction [66]. When STAG1/STAG2 is inhibited, the rings disassemble, resulting in the loss of cohesion. Later findings using budding yeast as a model supported that Scc3 or Pds5 may serve as factors that structurally stabilize two cohesin rings, forming a handcuff conformation [67, 68]. Notably, the removal of Pds5 in cells did not result in alterations in cohesin levels on chromatin, even though changes in sister chromatid cohesion were observed. Recent studies have also provided compelling evidence for the presence of cohesin dimers in cells [24, 65]. Cohesin dimers, which involve two cohesin molecules working together, appear to play a crucial role in the organization of chromosomes. Despite these significant findings, our understanding of cohesin dimers and their precise role in chromosomal organization is still evolving. More in-depth research is necessary to uncover the intricacies of cohesin dimerization and its impact on chromosome dynamics during cell division. Future investigations, such as reconstructing the human cohesin complex binding to chromatin in vitro and conducting structural and microscopy studies, as well as high-throughput chromosome conformation capture (Hi-C) experiments, are expected to provide additional insights into cohesin’s detailed role in regulating high-order chromatin structures and may reveal the true nature of cohesin rings in the context of cell division.

## Cohesin and accurate chromosome segregation

Accurate chromosome segregation during anaphase is a fundamental process that relies on the tight regulation of cohesin [69–71]. Cohesin maintains the connection from the moment sister chromatids are synthesized during the S phase until their separation in anaphase. During mitosis, the assembly of the spindle apparatus becomes an important event, as it engages the kinetochores of all chromosomes. Notably, the proper attachment of kinetochores to spindle microtubules, referred to as amphitelic (bi-orientation) attachment, is a key determinant in enabling the eventual separation of sister chromatids [72–74]. This separation is executed by the enzymatic cleavage of specific cohesin subunits, namely RAD21, catalyzed by the enzyme Separase. After cohesin cleavage, sister chromatids are released and move toward opposite poles due to the action of the spindle apparatus [6–8] (Fig. 1).

### Role of separase

It is important to recognize that the removal of cohesin, and consequently the separation of sister chromatids, is an irreversible step. Therefore, the removal of cohesin needs to be synchronized for the proper segregation of the chromatids to opposite spindle poles [8, 75]. Premature separation of sister chromatids can lead to aneuploidy, a condition characterized by an abnormal number of chromosomes, and potentially contribute to tumorigenesis [8, 10, 76, 77]. At the metaphase-to-anaphase transition, Separase, an evolutionarily conserved endopeptidase, cleaves RAD21 to separate the sister chromatids [6–8]. Separase activity is tightly regulated via several mechanisms to ensure timely cleavage of cohesin-RAD21 during the metaphase-to-anaphase transition [8, 78–82]. Separase is inactive when it binds to its inhibitory chaperon, securin. It is also inhibited via phosphorylation by Cyclin B-CDK1 kinase and binding to Cyclin B [81]. After activation at the onset of anaphase, the anaphase-promoting complex polyubiquitinates mitotic cyclin and securin, which are rapidly degraded by 26S proteasome. Once activated, Separase proteolytically cleaves the cohesin subunit RAD21.

### The consequences of cohesin dysregulation and Separase overexpression

Despite many control mechanisms, overexpression of Separase, a feature of many human tumors including breast, prostate, bone, brain, and blood cancers [33, 83–89], has been reported to cause chromosomal missegregation and aneuploidy [8, 33, 84, 90]. *ESPL1* is an oncogene, the transcription of which can be activated by the steroid hormones estrogen and progesterone [91], and that overexpression of Separase induces chromosomal missegregation in tissue culture, mammary transplants, and transgenic animals through premature separation of chromatids, resulting in aneuploidy and mammary tumorigenesis [33, 84]. The significance and relevance of these studies in mice are underscored by the finding that Separase is overexpressed in numerous human tumors.

Further, overexpression of Separase strongly correlates with a high incidence of relapse and metastasis and a lower 5-year overall survival rate [8, 83, 85, 90]. Meta-analysis indicates a strong positive correlation between Separase mRNA expression and tumor grade, and a strong negative correlation with disease-free and overall survival [8, 83, 85, 87–89, 92–94]. Despite its overexpression in numerous human tumors, the role of Separase as an oncogene and its relevance as an oncotarget have been grossly understudied [8]. Specifically, how Separase overexpression-driven aneuploidy overcomes the threshold of tumor-resisting forces within the cell and results in the initiation of tumor formation and how other cooperating lesions further this process need to be investigated.

### Cohesin mutations and aneuploidy

Mutations in cohesin have been a subject of intense research, and their relationship with aneuploidy has generated significant interest [77], [95]. Somatic mutations in genes associated with the cohesin complex and its regulators are prevalent in various cancer types, such as glioblastoma, Ewing’s sarcoma, bladder cancer, and myeloid neoplasms [96–112]. These mutations, which can range from 4 to 36% in different cancers, tend to involve genes encoding core cohesin subunits [71, 113–115]. Interestingly, these mutations typically occur in a mutually exclusive manner, without specific hotspots identified. Among the cohesin subunits, *STAG2* stands out as the most frequently mutated subunit and is implicated in four or more cancer types [111, 116]. Moreover, certain cases of myeloid neoplasms and Ewing sarcoma have revealed instances of reduced cohesin gene expression, even in the absence of mutations [105, 110]. In a parallel line of genetic alterations, mutations affecting the binding sites of cohesin and CTCF, a cohesin-associated protein, have been observed in multiple cancer types [117]. It is important to note that cohesin mutations alone are insufficient to drive malignancy. Rather, they must coincide with other genetic mutations and collaborate with abnormal signaling events to contribute to cancer progression [105, 106, 108, 112]. A recent study in budding yeast found that under normal conditions, cohesin’s role in maintaining cohesion between sister chromatids serves to suppress aneuploidy and prevent whole chromosome loss of heterozygosity. However, when sister chromatid cohesion becomes compromised, it can result in significant genetic consequences including whole chromosome loss of heterozygosity [118]. This underscores the cooperative and context-dependent nature of cohesin mutations in the development of cancer.

Several studies have explored the impact of cohesin mutations on aneuploidy, and while a clear connection exists in some cases, the relationship is more complex in others [77]. Cohesin deficiency in cell models can lead to chromosome and cell cycle irregularities. While cohesin mutations were initially expected to be linked with aneuploidy in cancers, the connection is not straightforward. Some studies found an association between cohesin mutations and chromosome copy-number variations, while others did not observe increased aneuploidy in cohesin mutant cancers, such as bladder cancer, Ewing sarcoma, or myeloid neoplasms. Interestingly, specific types of *STAG2* mutations adversely affected chromatid cohesion and chromosome segregation, but this did not consistently result in a substantial increase in chromosome number [119]. Reduced cohesin expression was also linked to chromosome instability in some cases, characterized by increased micronuclear formation and nuclear size [120]. In summary, cohesin mutations can lead to chromosome instability in certain cases, but this does not necessarily result in aneuploidy. Therefore, the link between cohesin mutations and aneuploidy is still debated.

Several studies suggest that the primary mechanism by which cohesin mutations contribute to cancer is through disrupting genome organization and transcription impacting proto-oncogenes or tumor suppressor genes [115, 121–131]. Dysfunctional cohesin affects its dynamic binding to chromatin and impairs the recruitment of RNA polymerase II, leading to transcriptional dysregulation. Cancer-associated mutations in cohesin genes can lead to aberrant DNA looping, dysregulation of crucial lineage-specific transcription factors, and misexpression of genes responsible for cellular identity and homeostasis [122, 125, 126, 131–136]. Cohesin insufficiency can enhance self-renewal while impairing differentiation, leading to abnormal cellular plasticity, which is central to malignant transformation. This effect has been observed in hematopoietic stem cells, where cohesin insufficiency results in misexpression of critical genes involved in hematopoiesis [122, 125, 126, 131–134]. In mouse embryonic stem cells, cohesin depletion reduces enhancer–promoter interactions at pluripotency genes, causing a loss of the pluripotent state [137, 138]. Loss-of-function mutations in *STAG2*, found in Ewing’s sarcoma, alter dynamic loop extrusion, decrease cis-promoter–enhancer interactions, and lead to significant changes in the transcriptome [139]. Replication stress, involving challenges to DNA replication fork progression, can be influenced by cohesin, which accumulates at stalled forks and facilitates template switching to repair DNA lesions [140]. Cohesin depletion increases Pol II pausing at cohesin-binding genes, highlighting its role in resolving replication stress [141]. STAG1 is also implicated in interactions with the Super Elongation Complex, a process linked to leukemia and multiple myeloma pathogenesis [142]. Overall, cohesin defects play a significant role in cancer development, involving a complex interplay of aneuploidy, altered gene expression, and participation in DNA replication and repair processes. However, distinguishing between aneuploidy and transcriptional changes resulting from cohesin mutations is experimentally challenging, suggesting that both processes likely occur concurrently.

In summary, the connection between cohesin mutations and aneuploidy is complex and context-dependent. While some cancers show a clear link between cohesin mutations and aneuploidy, others do not, emphasizing the multifaceted role of cohesin mutations in cancer development. Understanding the specifics of these relationships is crucial for developing targeted therapies and treatments for various cancer types.

## Aneuploidy and its link to cancer: an overview

Aneuploidy is a hallmark of human cancers [12, 143, 144]. It refers to an imbalanced karyotype marked by an abnormal number of chromosomes in cells. The frequency of aneuploidy varies among different cancer types, ranging from approximately 25% in thyroid carcinomas to nearly 100% in glioblastomas [145]. About 97% of breast cancers show one or more aneuploidies, where around 60% of breast cancer tumors contain an extra copy of chromosome arm 1q11, which is a more prevalent alteration compared to mutations in genes like *PIK3CA* (found in 39% of breast tumors) that encodes Phosphatidylinositol-4,5-Bisphosphate 3-Kinase Catalytic Subunit Alpha, or *TP53* (in 34%) [145] which encodes p53 protein, a tumor suppressor. Aneuploidy’s exact causes remain unclear, but it can indirectly lead to changes in gene expression from trisomic and monosomic chromosomes. In contrast, healthy, non-cancerous cells in the human body are typically very strict about maintaining the right number and structure of chromosomes during cell division. This key difference between cancer cells and normal cells has given rise to the hypothesis that cancer cells have somehow developed the ability to overcome inherent barriers to changes in chromosomes that normal cells cannot tolerate. This means that they have acquired unique strategies to deal with these genetic alterations. However, what this strategy is currently remain undefined.

### Aneuploidy’s dual role: neither universal oncogenic nor tumor suppressor

Aneuploidy frequently develops early in tumorigenesis [146–151] with some chromosomal alterations occurring later in tumor development, potentially after cells have acquired mutations allowing them to tolerate aneuploidy’s effects [152]. On the other hand, considering that in most cases, aneuploidy can be detrimental to cell fitness, it is possible that aneuploidy serves as an early evolutionary response to counteract uncontrolled cell division triggered by factors, such as oncogenic mutations, epigenetic changes, or environmental stress. The fact that many aneuploidies are capable of functioning as tumor suppressors [12] supports this alternate hypothesis. However, as cells face repeated oncogenic challenges, they may adapt to tolerate aneuploidy through genetic or epigenetic alterations that support uncontrolled growth. Evidence for this concept comes from observations that aneuploid cells can acquire mutations enhancing their proliferative capacity, such as in the ubiquitin–proteasome pathway of budding yeast [153]. In mammalian cells, the loss of the tumor suppressor p53 has been shown to facilitate the growth of aneuploid cells [12, 154, 155]. In a model of hormone-induced spontaneous tumorigenesis in p53-null mammary glands, our research has revealed that the rate of chromosome missegregation can be significantly influenced by changes in the levels of two crucial proteins: Separase and MAD2, which play essential roles in maintaining proper chromosomal segregation and the spindle checkpoint during mitosis [91]. As tumor progresses, cells counter-evolve and further adapt to tolerate aneuploidy through significant chromosomal and genomic alterations, enabling them to meet the demands of uncontrolled tumor growth.

The precise mechanisms governing aneuploidy tolerance and the shift from aneuploidy’s anti-tumorigenic traits to cancer-promoting features remain elusive. The ways in which cancer cells maintain vigorous growth despite highly aneuploid karyotypes are still actively under investigation and lack a comprehensive understanding. In some rare instances, aneuploid karyotypes may exhibit properties that benefit cancer development, such as immune evasion, drug resistance, and the overexpression of oncogenes. These advantageous aneuploidies can gradually become more prevalent within the tumor as they are selectively favored over time [12].

### Chromosomal instability and aneuploidy

Chromosomal instability (CIN) and aneuploidy are intricately connected in cancer [156, 157]. While one can be considered the cause, the other is the resulting effect. Aneuploidy characterizes an unbalanced chromosomal arrangement, whereas CIN is a state in which cells frequently missegregate whole chromosomes, thus playing a role in the development of aneuploidy [156, 158]. While CIN can give rise to aneuploidy, aneuploidy can lead to CIN. Similarly, like that of aneuploidy, CIN can also exert both tumor-promoting and tumor-suppressive effects [12]. The mechanisms of chromosomal instability have been extensively covered in several excellent reviews [159–162], but the outcome is consistently the missegregation of chromosomes. Missegregated chromosomes do not always result in aneuploidy but can trigger various other effects. These include DNA damage, activation of the cytosolic DNA-sensing pathway involving cyclic GMP–AMP synthase (cGAS) and stimulator of interferon genes (STING), and chromothripsis, a phenomenon where one or more chromosomes shatter and are incorrectly reassembled [163–167]. Hence, these CIN can impact tumorigenesis through mechanisms that are independent of aneuploidy.

### Consequences of aneuploidy

Aneuploidy, through the gain or loss of whole chromosomes, creates imbalances in the proteome [162, 168], particularly in proteins crucial for DNA metabolism and cell cycle regulation, elevating the risk of DNA mutations [169–171]. Single-chromosomal aneuploidy in budding yeast producing a modest, but significant, elevation in the rates of point mutations and mitotic recombination supports this notion [172]. Aneuploidy-driven genome instability could arise from chromosome missegregation errors in mammalian cells, which leads to double-strand breaks as a result of lagging anaphase chromosomes trapped in the cleavage furrow during cytokinesis [10, 173]. In addition to the damage caused directly by the mitotic machinery, lagging chromosomes, including those that are not missegregated, often form micronuclei, which also accumulate high levels of DNA damage resulting in chromosome fragmentations or shattering leading to chromothripsis [10, 174–178]. While epigenetic dysregulation is recognized as a factor in cancer [179, 180], its connection to aneuploidy remains a less-explored area.

Aneuploidy can influence the behavior of oncogenes and tumor suppressors without triggering an immune response. Additionally, aneuploidy can change the expression of antigens and cell surface markers, creating a diverse range that helps certain cells evade immune detection. As indicated earlier, a related feature, chromosomal instability, can activate pathways like cGAS-STING, leading to a phenomenon called the senescence-associated secretory phenotype (SASP) [181]. While SASP can aid the immune system, particularly natural killer cells, in recognizing and eliminating aneuploid cells, it can also contribute to tumor-promoting behaviors such as mesenchymal transitions, chronic inflammation, and the release of factors that support tumor growth [12]. This intricate relationship between aneuploidy, the immune system, and tumorigenesis is currently a subject of active investigation.

Aneuploidy can arise from various cellular defects, including issues with the spindle apparatus, centrosome and centriole abnormalities, checkpoint maintenance, and altered telomere and centromere stability [10]. These defects can occur spontaneously or be induced by mutations in relevant genes, potentially causing aneuploidy. Recent studies demonstrate that aneuploidy alone can trigger mutations that drive cancer initiation and progression [156, 182, 183]. However, the full spectrum of mutations and epigenetic changes associated with aneuploidy in the context of tumor development remains a subject of ongoing investigation.

## Aneuploidy and cancer therapy: a promising avenue

Surprisingly, despite being a consistent feature of human tumors, aneuploidy status has not been harnessed for cancer therapy. Aneuploidy, a common feature in various cancers but rare in normal tissues, presents a promising target for cancer treatment. This distinction between cancerous and healthy tissues holds the potential for a captivating avenue in cancer therapy, where aneuploid cancer cells could be selectively targeted while sparing normal, diploid cells, thus reducing side effects. This innovative approach holds promise for a wide spectrum of cancer types [184].

One avenue of exploration is to exploit the inherent vulnerability of untransformed aneuploid cells to certain metabolic stressors. Studies have shown that aneuploid cells are more sensitive to the activation of the AMP-activated protein kinase (AMPK) and peroxisome proliferator-activated receptor-γ co-activator 1α (PGC1α) pathway [185, 186]. This pathway plays a central role in regulating energy metabolism and mitochondrial biogenesis. Aneuploid cells are already under considerable energy stress, and further disruption of these metabolic pathways may push them over the edge, causing toxic levels of metabolic dysregulation. Additionally, aneuploid cells have higher levels of ceramide, a type of lipid molecule, and further elevating ceramide levels is significantly more toxic to aneuploid cells compared to diploid cells [187].

Another approach to targeting aneuploid cancer cells is to exacerbate their chromosomal mis-segregation [188]. Since aneuploidy and chromosomal instability (CIN) are closely intertwined, increasing the rate of chromosome missegregation beyond tolerable levels may specifically compromise the viability of aneuploid cells. Many aneuploid cancer cells exhibit altered microtubule dynamics and increased stability of microtubule–kinetochore connections. These spindle disruptions can result in improper kinetochore attachments, hinder error correction mechanisms, and lead to high levels of chromosome missegregation. Inhibiting the spindle-assembly checkpoint has been shown to selectively kill aneuploid cancer cells by further enhancing their level of CIN [189–192].

Furthermore, recent research suggests that aneuploid cancer cells may be sensitive to spindle disruption by inhibiting a mitotic kinesin, even without a complete loss of the spindle-assembly checkpoint [192]. Specifically, the mitotic kinesin KIF18A, which is generally dispensable for most mammalian cell proliferation, appears to be more toxic to aneuploid cells compared to diploid cells [193, 194]. Knockdown of *KIF18A* in cells with highly aneuploid karyotypes leads to alterations in spindle geometry and microtubule dynamics, resulting in mitotic errors, micronucleus formation, and a reduction in cellular viability.

These findings suggest that KIF18A inhibition could hold promise as a target for future drug development aimed at establishing anti-aneuploidy therapeutic strategies for cancer treatment [195]. Overall, these approaches offer exciting possibilities for selectively targeting aneuploid cancer cells, providing new avenues for developing effective and targeted cancer therapies.

## Separase: an innovative target to tackle aneuploidy

During cell division, Separase’s enzymatic activity is brief and requires minimal active enzyme levels to cleave cohesin molecules [50, 196]. Therefore, unlike other mitotic targets, pharmacologic inhibition of Separase by small molecule drugs presents an effective strategy to be a more effective mitotic target in inhibiting the proliferation of cancer cells addicted to Separase overexpression without affecting the normal cell division [8].

Separase, an oncogene overexpressed in multiple cancers, has been linked to aneuploidy and tumorigenesis [33, 83, 85–89]. Premature chromatid segregation due to overactive Separase can lead to aneuploidy. Its conditional activation in mouse mammary epithelial cells induces gross aneuploidy and mammary tumors that are reversible upon removal of activation [33, 84]. Over 60% of human breast cancer tumors overexpress Separase, and its expression correlates with poor prognosis and altered tumor subtypes [33, 83]. In mouse model, Separase overexpression promotes aneuploidy and genetic heterogeneity, leading to mammary tumorigenesis [84]. Homozygous deletion of *ESPL1*, the gene encoding Separase, results in early embryonic lethality in mice [197–199]. In contrast, *ESPL1* heterozygote mice with significantly lower Separase levels exhibit no apparent phenotype. Using a hypomorphic Separase mouse model, researchers observed a cancer-free phenotype and increased lifespan compared to wild-type mice [197–199]. *ESPL1* overexpression, on the other hand, leads to aneuploidy and tumorigenesis [33, 85]. Therefore, pharmacologically reducing Separase activity in cancer cells offers a new approach to combating aneuploidy and tumorigenesis [8].

Knockdown of Separase inhibits the growth of breast cancer cells while sparing normal mammary epithelial cells. Small molecule inhibitors of Separase, known as Sepins, have been identified and shown to be effective in selectively targeting Separase-overexpressing cancer cells in culture and in xenograft studies [200]. Therefore, Separase represents an ideal therapeutic target to eliminate Separase-overexpressed aneuploid tumors including various breast tumor subtypes, and Sepins offer a promising avenue for breast and other cancer therapeutics.

## Conclusion and future prospective

To conclude, the intricate processes of chromosomal cohesion and separation play pivotal roles in governing genomic integrity and fidelity, making them key players in the complex landscape of cancer biology. Any malfunction in this process can result in chromosomal anomalies, leading to abnormal chromosome content, a condition known as aneuploidy. Aneuploidy is a defining characteristic of human tumors and contributes significantly to the initiation and progression of cancer. Overexpression of Separase has been linked to chromosomal missegregation, aneuploidy, and tumorigenesis. Thus, the level of Separase activity is a crucial determinant of genomic stability. Notably, Separase has emerged as an innovative therapeutic target to tackle aneuploidy in cancer. Small molecule inhibitors of Separase, known as Sepins, show promise in selectively targeting Separase-overexpressing cancer cells while sparing normal cells. This targeted approach offers a novel avenue for cancer therapy, potentially reducing side effects and improving the prognosis for various cancer types, particularly those with high Separase expression.

The intricate relationship between aneuploidy, chromosomal instability, and cancer also remains a topic of ongoing investigation, offering a fertile ground for future research. As we uncover more details about the mechanisms and consequences of aneuploidy in cancer, we move closer to the development of innovative and targeted therapies that could improve the prognosis for a wide range of cancer types.

In summary, the roles of chromosomal cohesion and separation in the context of aneuploidy and cancer are tightly intertwined. The precise regulation of these processes is essential to maintain genomic stability, and their dysregulation can lead to aneuploidy, a hallmark of cancer. By targeting key players like Separase, we may pave the way for innovative and effective cancer therapies that specifically address the unique vulnerabilities of aneuploid cancer cells, offering hope for improved cancer treatment outcomes.

## Conflict of interest

The author declare no competing interests.

## Data Availability

There is no data or material to share.

## References

[1] GM C (2000) The Cell: A Molecular Approach, Edn. 2nd. (Sunderland (MA): Sinauer Associates (Oxford University Press), MA

[2] Nasmyth K, Haering CH (2005). The structure and function of SMC and kleisin complexes. Annu Rev Biochem.

[3] Hirano T (2000). Chromosome cohesion, condensation, and separation. Annu Rev Biochem.

[4] Koshland DE, Guacci V (2000). Sister chromatid cohesion: the beginning of a long and beautiful relationship. Curr Opin Cell Biol.

[5] Nasmyth K, Peters JM, Uhlmann F (2000). Splitting the chromosome: cutting the ties that bind sister chromatids. Science.

[6] Uhlmann F, Lottspeich F, Nasmyth K (1999). Sister-chromatid separation at anaphase onset is promoted by cleavage of the cohesin subunit Scc1. Nature.

[7] Waizenegger IC, Hauf S, Meinke A, Peters JM (2000). Two distinct pathways remove mammalian cohesin from chromosome arms in prophase and from centromeres in anaphase. Cell.

[8] Zhang N, Pati D (2017). Biology and insights into the role of cohesin protease separase in human malignancies. Biol Rev Camb Philos Soc.

[9] Duesberg P, Fabarius A, Hehlmann R (2004). Aneuploidy, the primary cause of the multilateral genomic instability of neoplastic and preneoplastic cells. IUBMB Life.

[10] Holland AJ, Cleveland DW (2012). Losing balance: the origin and impact of aneuploidy in cancer. EMBO Rep.

[11] Santaguida S, Amon A (2015). Short- and long-term effects of chromosome mis-segregation and aneuploidy. Nat Rev Mol Cell Biol.

[12] Vasudevan A (2021). Aneuploidy as a promoter and suppressor of malignant growth. Nat Rev Cancer.

[13] Murray AW, Szostak JW (1985). Chromosome segregation in mitosis and meiosis. Annu Rev Cell Biol.

[14] Koshland D, Hartwell LH (1987). The structure of sister minichromosome DNA before anaphase in Saccharomyces cerevisiae. Science.

[15] Guacci V, Koshland D, Strunnikov A (1997). A direct link between sister chromatid cohesion and chromosome condensation revealed through the analysis of MCD1 in *S. cerevisiae*. Cell.

[16] Michaelis C, Ciosk R, Nasmyth K (1997). Cohesins: chromosomal proteins that prevent premature separation of sister chromatids. Cell.

[17] Arumugam P, Nishino T, Haering CH, Gruber S, Nasmyth K (2006). Cohesin’s ATPase activity is stimulated by the C-terminal Winged-Helix domain of its kleisin subunit. Curr Biol.

[18] Haering CH, Lowe J, Hochwagen A, Nasmyth K (2002). Molecular architecture of SMC proteins and the yeast cohesin complex. Mol Cell.

[19] Haering CH (2004). Structure and stability of cohesin’s Smc1-kleisin interaction. Mol Cell.

[20] Gligoris TG (2014). Closing the cohesin ring: structure and function of its Smc3-kleisin interface. Science.

[21] Gruber S, Haering CH, Nasmyth K (2003). Chromosomal cohesin forms a ring. Cell.

[22] Shi Z, Gao H, Bai XC, Yu H (2020). Cryo-EM structure of the human cohesin-NIPBL-DNA complex. Science.

[23] Cheng H, Zhang N, Pati D (2020). Cohesin subunit RAD21: from biology to disease. Gene.

[24] Shi D (2020). The acetyltransferase Eco1 elicits cohesin dimerization during S phase. J Biol Chem.

[25] Cuadrado A, Losada A (2020). Specialized functions of cohesins STAG1 and STAG2 in 3D genome architecture. Curr Opin Genet Dev.

[26] Pan H (2020). Cohesin SA1 and SA2 are RNA binding proteins that localize to RNA containing regions on DNA. Nucleic Acids Res.

[27] Ciosk R (2000). Cohesin’s binding to chromosomes depends on a separate complex consisting of Scc2 and Scc4 proteins. Mol Cell.

[28] Sumara I, Vorlaufer E, Gieffers C, Peters BH, Peters JM (2000). Characterization of vertebrate cohesin complexes and their regulation in prophase. J Cell Biol.

[29] Losada A, Yokochi T, Kobayashi R, Hirano T (2000). Identification and characterization of SA/Scc3p subunits in the Xenopus and human cohesin complexes. J Cell Biol.

[30] Ivanov D (2002). Eco1 is a novel acetyltransferase that can acetylate proteins involved in cohesion. Curr Biol.

[31] Watrin E (2006). Human Scc4 is required for cohesin binding to chromatin, sister-chromatid cohesion, and mitotic progression. Curr Biol.

[32] Onn I, Heidinger-Pauli JM, Guacci V, Unal E, Koshland DE (2008). Sister chromatid cohesion: a simple concept with a complex reality. Annu Rev Cell Dev Biol.

[33] Zhang N (2008). Overexpression of separase induces aneuploidy and mammary tumorigenesis. Proc Natl Acad Sci U S A.

[34] Zhang N, Panigrahi AK, Mao Q, Pati D (2011). Interaction of Sororin protein with polo-like kinase 1 mediates resolution of chromosomal arm cohesion. J Biol Chem.

[35] Nasmyth K (2011). Cohesin: a catenase with separate entry and exit gates?. Nat Cell Biol.

[36] Zhang N, Pati D (2012). Sororin is a master regulator of sister chromatid cohesion and separation. Cell Cycle.

[37] Kitajima TS (2006). Shugoshin collaborates with protein phosphatase 2A to protect cohesin. Nature.

[38] Wendt KS (2017). Resolving the genomic localization of the kollerin cohesin-loader complex. Methods Mol Biol.

[39] Shintomi K, Hirano T (2009). Releasing cohesin from chromosome arms in early mitosis: opposing actions of Wapl-Pds5 and Sgo1. Genes Dev.

[40] Wutz G (2017). Topologically associating domains and chromatin loops depend on cohesin and are regulated by CTCF, WAPL, and PDS5 proteins. EMBO J.

[41] Zhang N, Coutinho LE, Pati D (2021). PDS5A and PDS5B in cohesin function and human disease. Int J Mol Sci.

[42] Zhang J (2008). Acetylation of Smc3 by Eco1 is required for S phase sister chromatid cohesion in both human and yeast. Mol Cell.

[43] Wutz G (2020). ESCO1 and CTCF enable formation of long chromatin loops by protecting cohesin(STAG1) from WAPL. Elife.

[44] van der Lelij P (2009). The cellular phenotype of Roberts syndrome fibroblasts as revealed by ectopic expression of ESCO2. PLoS ONE.

[45] Deardorff MA (2012). HDAC8 mutations in Cornelia de Lange syndrome affect the cohesin acetylation cycle. Nature.

[46] Beckouet F (2010). An Smc3 acetylation cycle is essential for establishment of sister chromatid cohesion. Mol Cell.

[47] Borges V (2010). Hos1 deacetylates Smc3 to close the cohesin acetylation cycle. Mol Cell.

[48] Hauf S (2005). Dissociation of cohesin from chromosome arms and loss of arm cohesion during early mitosis depends on phosphorylation of SA2. PLoS Biol.

[49] Uhlmann F (2001). Secured cutting: controlling separase at the metaphase to anaphase transition. EMBO Rep.

[50] Hauf S, Waizenegger IC, Peters JM (2001). Cohesin cleavage by separase required for anaphase and cytokinesis in human cells. Science.

[51] Beauchene NA (2010). Rad21 is required for centrosome integrity in human cells independently of its role in chromosome cohesion. Cell Cycle.

[52] Schockel L, Mockel M, Mayer B, Boos D, Stemmann O (2011). Cleavage of cohesin rings coordinates the separation of centrioles and chromatids. Nat Cell Biol.

[53] Tsou MF (2009). Polo kinase and separase regulate the mitotic licensing of centriole duplication in human cells. Dev Cell.

[54] Diaz-Martinez LA (2010). Cohesin is needed for bipolar mitosis in human cells. Cell Cycle.

[55] Nasmyth K, Haering CH (2009). Cohesin: its roles and mechanisms. Annu Rev Genet.

[56] Mehta GD, Kumar R, Srivastava S, Ghosh SK (2013). Cohesin: functions beyond sister chromatid cohesion. FEBS Lett.

[57] Zhang N (2008). A handcuff model for the cohesin complex. J Cell Biol.

[58] Zhang N, Pati D (2009). Handcuff for sisters: a new model for sister chromatid cohesion. Cell Cycle.

[59] Xiang S, Koshland D (2021) Cohesin architecture and clustering in vivo. Elife 10 10.7554/eLife.62243 PMC7932697 33594972

[60] Matityahu A, Onn I (2022). It’s all in the numbers: Cohesin stoichiometry. Front Mol Biosci.

[61] Nasmyth K, Schleiffer A (2004). From a single double helix to paired double helices and back. Philos Trans R Soc Lond B Biol Sci.

[62] Ivanov D, Nasmyth K (2005). A topological interaction between cohesin rings and a circular minichromosome. Cell.

[63] Haering CH, Farcas AM, Arumugam P, Metson J, Nasmyth K (2008). The cohesin ring concatenates sister DNA molecules. Nature.

[64] Eng T, Guacci V, Koshland D (2015). Interallelic complementation provides functional evidence for cohesin-cohesin interactions on DNA. Mol Biol Cell.

[65] Gutierrez-Escribano P (2019). A conserved ATP- and Scc2/4-dependent activity for cohesin in tethering DNA molecules. Sci Adv.

[66] Zhang N (2013). Characterization of the interaction between the cohesin subunits Rad21 and SA1/2. PLoS ONE.

[67] Kulemzina I (2012). Cohesin rings devoid of Scc3 and Pds5 maintain their stable association with the DNA. PLoS Genet.

[68] Tong K, Skibbens RV (2015). Pds5 regulators segregate cohesion and condensation pathways in Saccharomyces cerevisiae. Proc Natl Acad Sci U S A.

[69] Peters JM, Tedeschi A, Schmitz J (2008). The cohesin complex and its roles in chromosome biology. Genes Dev.

[70] Haarhuis JH, Elbatsh AM, Rowland BD (2014). Cohesin and its regulation: on the logic of X-shaped chromosomes. Dev Cell.

[71] Di Nardo M, Pallotta MM, Musio A (2022). The multifaceted roles of cohesin in cancer. J Exp Clin Cancer Res.

[72] Musacchio A (2015). The molecular biology of spindle assembly checkpoint signaling dynamics. Curr Biol.

[73] Foley EA, Kapoor TM (2013). Microtubule attachment and spindle assembly checkpoint signalling at the kinetochore. Nat Rev Mol Cell Biol.

[74] Lara-Gonzalez P, Pines J, Desai A (2021). Spindle assembly checkpoint activation and silencing at kinetochores. Semin Cell Dev Biol.

[75] Konecna M, Abbasi Sani S, Anger M (2023). Separase and roads to disengage sister chromatids during anaphase. Int J Mol Sci.

[76] Losada A (2014). Cohesin in cancer: chromosome segregation and beyond. Nat Rev Cancer.

[77] Waldman T (2020). Emerging themes in cohesin cancer biology. Nat Rev Cancer.

[78] Stemmann O, Zou H, Gerber SA, Gygi SP, Kirschner MW (2001). Dual inhibition of sister chromatid separation at metaphase. Cell.

[79] Ciosk R (1998). An ESP1/PDS1 complex regulates loss of sister chromatid cohesion at the metaphase to anaphase transition in yeast. Cell.

[80] Gorr IH, Boos D, Stemmann O (2005). Mutual inhibition of separase and Cdk1 by two-step complex formation. Mol Cell.

[81] Hellmuth S (2015). Positive and negative regulation of vertebrate separase by Cdk1-cyclin B1 may explain why securin is dispensable. J Biol Chem.

[82] Hellmuth S (2015). Human chromosome segregation involves multi-layered regulation of separase by the peptidyl-prolyl-isomerase Pin1. Mol Cell.

[83] Meyer R (2009). Overexpression and mislocalization of the chromosomal segregation protein separase in multiple human cancers. Clin Cancer Res.

[84] Mukherjee M (2014). MMTV-Espl1 transgenic mice develop aneuploid, estrogen receptor alpha (ERalpha)-positive mammary adenocarcinomas. Oncogene.

[85] Mukherjee M (2014). Overexpression and constitutive nuclear localization of cohesin protease Separase protein correlates with high incidence of relapse and reduced overall survival in glioblastoma multiforme. J Neurooncol.

[86] Finetti P (2014). ESPL1 is a candidate oncogene of luminal B breast cancers. Breast Cancer Res Treat.

[87] Gurvits N (2017). Separase is a marker for prognosis and mitotic activity in breast cancer. Br J Cancer.

[88] Repo H (2020). A prognostic model based on cell-cycle control predicts outcome of breast cancer patients. BMC Cancer.

[89] Liu Z (2021). ESPL1 is a novel prognostic biomarker associated with the malignant features of glioma. Front Genet.

[90] Pati D (2008). Oncogenic activity of separase. Cell Cycle.

[91] Pati D (2004). Hormone-induced chromosomal instability in p53-null mammary epithelium. Cancer Res.

[92] Yang Q, Yu B, Sun J (2020). TTK, CDC25A, and ESPL1 as prognostic biomarkers for endometrial cancer. Biomed Res Int.

[93] Dawood S (2011). Defining breast cancer prognosis based on molecular phenotypes: results from a large cohort study. Breast Cancer Res Treat.

[94] He X, Zhang C, Shi C, Lu Q (2018). Meta-analysis of mRNA expression profiles to identify differentially expressed genes in lung adenocarcinoma tissue from smokers and non-smokers. Oncol Rep.

[95] Barbero JL (2011). Sister chromatid cohesion control and aneuploidy. Cytogenet Genome Res.

[96] Kandoth C (2013). Mutational landscape and significance across 12 major cancer types. Nature.

[97] Brennan CW (2013). The somatic genomic landscape of glioblastoma. Cell.

[98] Balbas-Martinez C (2013). Recurrent inactivation of STAG2 in bladder cancer is not associated with aneuploidy. Nat Genet.

[99] Guo G (2013). Whole-genome and whole-exome sequencing of bladder cancer identifies frequent alterations in genes involved in sister chromatid cohesion and segregation. Nat Genet.

[100] Solomon DA (2013). Frequent truncating mutations of STAG2 in bladder cancer. Nat Genet.

[101] Kon A (2013). Recurrent mutations in multiple components of the cohesin complex in myeloid neoplasms. Nat Genet.

[102] Yoshida K (2013). The landscape of somatic mutations in Down syndrome-related myeloid disorders. Nat Genet.

[103] Lawrence MS (2014). Discovery and saturation analysis of cancer genes across 21 tumour types. Nature.

[104] Hong CS (2020). Persistent STAG2 mutation despite multimodal therapy in recurrent pediatric glioblastoma. NPJ Genom Med.

[105] Crompton BD (2014). The genomic landscape of pediatric Ewing sarcoma. Cancer Discov.

[106] Tirode F (2014). Genomic landscape of Ewing sarcoma defines an aggressive subtype with co-association of STAG2 and TP53 mutations. Cancer Discov.

[107] Taylor CF, Platt FM, Hurst CD, Thygesen HH, Knowles MA (2014). Frequent inactivating mutations of STAG2 in bladder cancer are associated with low tumour grade and stage and inversely related to chromosomal copy number changes. Hum Mol Genet.

[108] Cancer Genome Atlas Research, N (2014). Comprehensive molecular characterization of urothelial bladder carcinoma. Nature.

[109] Thol F (2014). Mutations in the cohesin complex in acute myeloid leukemia: clinical and prognostic implications. Blood.

[110] Thota S (2014). Genetic alterations of the cohesin complex genes in myeloid malignancies. Blood.

[111] Leiserson MD (2015). Pan-cancer network analysis identifies combinations of rare somatic mutations across pathways and protein complexes. Nat Genet.

[112] Papaemmanuil E (2016). Genomic classification and prognosis in acute myeloid leukemia. N Engl J Med.

[113] De Koninck M, Losada A (2016). Cohesin mutations in cancer. Cold Spring Harb Perspect Med.

[114] Sondka Z (2018). The COSMIC Cancer Gene Census: describing genetic dysfunction across all human cancers. Nat Rev Cancer.

[115] Antony J, Chin CV, Horsfield JA (2021). Cohesin mutations in cancer: emerging therapeutic targets. Int J Mol Sci.

[116] Hill VK, Kim JS, Waldman T (2016). Cohesin mutations in human cancer. Biochim Biophys Acta.

[117] Katainen R (2015). CTCF/cohesin-binding sites are frequently mutated in cancer. Nat Genet.

[118] Sagi D, Marcos-Hadad E, Bari VK, Resnick MA, Covo S (2017). Increased LOH due to defective sister chromatid cohesion is due primarily to chromosomal aneuploidy and not recombination. G3 (Bethesda).

[119] Kim JS (2016). Intact cohesion, anaphase, and chromosome segregation in human cells harboring tumor-derived mutations in STAG2. PLoS Genet.

[120] Leylek TR, Jeusset LM, Lichtensztejn Z, McManus KJ (2020). Reduced expression of genes regulating cohesion induces chromosome instability that may promote cancer and impact patient outcomes. Sci Rep.

[121] Kojic A (2018). Distinct roles of cohesin-SA1 and cohesin-SA2 in 3D chromosome organization. Nat Struct Mol Biol.

[122] Viny AD (2019). Cohesin members Stag1 and Stag2 display distinct roles in chromatin accessibility and topological control of HSC self-renewal and differentiation. Cell Stem Cell.

[123] Antony J (2020). BET inhibition prevents aberrant RUNX1 and ERG transcription in STAG2 mutant leukaemia cells. J Mol Cell Biol.

[124] Antony J (2015). Cohesin modulates transcription of estrogen-responsive genes. Biochim Biophys Acta.

[125] Viny AD (2015). Dose-dependent role of the cohesin complex in normal and malignant hematopoiesis. J Exp Med.

[126] Mullenders J (2015). Cohesin loss alters adult hematopoietic stem cell homeostasis, leading to myeloproliferative neoplasms. J Exp Med.

[127] Horsfield JA (2007). Cohesin-dependent regulation of Runx genes. Development.

[128] Leeke B, Marsman J, O’Sullivan JM, Horsfield JA (2014). Cohesin mutations in myeloid malignancies: underlying mechanisms. Exp Hematol Oncol.

[129] Yun J (2016). Dynamic cohesin-mediated chromatin architecture controls epithelial-mesenchymal plasticity in cancer. EMBO Rep.

[130] Yun J (2016). Reduced cohesin destabilizes high-level gene amplification by disrupting pre-replication complex bindings in human cancers with chromosomal instability. Nucleic Acids Res.

[131] Mazumdar C (2015). Leukemia-associated cohesin mutants dominantly enforce stem cell programs and impair human hematopoietic progenitor differentiation. Cell Stem Cell.

[132] Kumar P (2023). Cohesin subunit RAD21 Regulates the differentiation and self-renewal of hematopoietic stem and progenitor cells. Stem Cells.

[133] Kumar P (2020). Haploinsufficiency of cohesin protease, separase, promotes regeneration of hematopoietic stem cells in mice. Stem Cells.

[134] Cuartero S (2018). Control of inducible gene expression links cohesin to hematopoietic progenitor self-renewal and differentiation. Nat Immunol.

[135] Galeev R (2016). Genome-wide RNAi screen identifies cohesin genes as modifiers of renewal and differentiation in human HSCs. Cell Rep.

[136] Sasca D (2019). Cohesin-dependent regulation of gene expression during differentiation is lost in cohesin-mutated myeloid malignancies. Blood.

[137] Rittenhouse NL et al (2021) Functional impact of cancer-associated cohesin variants on gene expression and cellular identity. Genetics 217 10.1093/genetics/iyab025 PMC8049558 33704438

[138] Nitzsche A (2011). RAD21 cooperates with pluripotency transcription factors in the maintenance of embryonic stem cell identity. PLoS ONE.

[139] Surdez D (2021). STAG2 mutations alter CTCF-anchored loop extrusion, reduce cis-regulatory interactions and EWSR1-FLI1 activity in Ewing sarcoma. Cancer Cell.

[140] van Schie JJM, de Lange J (2021). The interplay of cohesin and the replisome at processive and stressed DNA replication forks. Cells.

[141] Schaaf CA (2013). Genome-wide control of RNA polymerase II activity by cohesin. PLoS Genet.

[142] Izumi K (2015). Germline gain-of-function mutations in AFF4 cause a developmental syndrome functionally linking the super elongation complex and cohesin. Nat Genet.

[143] Weaver BA, Cleveland DW (2006). Does aneuploidy cause cancer?. Curr Opin Cell Biol.

[144] Gordon DJ, Resio B, Pellman D (2012). Causes and consequences of aneuploidy in cancer. Nat Rev Genet.

[145] Taylor AM (2018). Genomic and functional approaches to understanding cancer aneuploidy. Cancer Cell.

[146] Aldaz CM, Conti CJ, Klein-Szanto AJ, Slaga TJ (1987). Progressive dysplasia and aneuploidy are hallmarks of mouse skin papillomas: relevance to malignancy. Proc Natl Acad Sci U S A.

[147] Aldaz CM, Trono D, Larcher F, Slaga TJ, Conti CJ (1989). Sequential trisomization of chromosomes 6 and 7 in mouse skin premalignant lesions. Mol Carcinog.

[148] Garewal HS, Sampliner R, Liu Y, Trent JM (1989). Chromosomal rearrangements in Barrett’s esophagus. A premalignant lesion of esophageal adenocarcinoma. Cancer Genet Cytogenet.

[149] Longy M (1990). Chromosomal analysis of colonic adenomatous polyps. Cancer Genet Cytogenet.

[150] Crowell RE (1996). Detection of trisomy 7 in nonmalignant bronchial epithelium from lung cancer patients and individuals at risk for lung cancer. Cancer Epidemiol Biomarkers Prev.

[151] Mian C (1999). Fluorescence in situ hybridization in cervical smears: detection of numerical aberrations of chromosomes 7, 3, and X and relationship to HPV infection. Gynecol Oncol.

[152] Gerstung M (2020). The evolutionary history of 2,658 cancers. Nature.

[153] Torres EM (2010). Identification of aneuploidy-tolerating mutations. Cell.

[154] Thompson S, Compton DA (2010). Proliferation of aneuploid human cells is limited by a p53-dependent mechanism. J Cell Biol.

[155] Soto M (2017). p53 Prohibits Propagation of Chromosome Segregation Errors that Produce Structural Aneuploidies. Cell Rep.

[156] Giam M, Rancati G (2015). Aneuploidy and chromosomal instability in cancer: a jackpot to chaos. Cell Div.

[157] van Jaarsveld RH, Kops G (2016). Difference makers: chromosomal instability versus aneuploidy in cancer. Trends Cancer.

[158] Schukken KM, Foijer F (2018) CIN and aneuploidy: different concepts, different consequences. Bioessays 40 10.1002/bies.201700147 29160563

[159] Thompson SL, Bakhoum SF, Compton DA (2010). Mechanisms of chromosomal instability. Curr Biol.

[160] Dhital B, Rodriguez-Bravo V (2023). Mechanisms of chromosomal instability (CIN) tolerance in aggressive tumors: surviving the genomic chaos. Chromosome Res.

[161] Gollin SM (2004). Chromosomal instability. Curr Opin Oncol.

[162] Pfau SJ, Amon A (2012). Chromosomal instability and aneuploidy in cancer: from yeast to man. EMBO Rep.

[163] Zhang CZ (2015). Chromothripsis from DNA damage in micronuclei. Nature.

[164] Mackenzie KJ (2017). cGAS surveillance of micronuclei links genome instability to innate immunity. Nature.

[165] de Oliveira Mann CC, Kranzusch PJ (2017). cGAS conducts micronuclei DNA surveillance. Trends Cell Biol.

[166] Bakhoum SF, Cantley LC (2018). The multifaceted role of chromosomal instability in cancer and its microenvironment. Cell.

[167] Bakhoum SF (2018). Chromosomal instability drives metastasis through a cytosolic DNA response. Nature.

[168] Pavelka N (2010). Aneuploidy confers quantitative proteome changes and phenotypic variation in budding yeast. Nature.

[169] Duesberg P (1999). How aneuploidy may cause cancer and genetic instability. Anticancer Res.

[170] Loeb LA (2001). A mutator phenotype in cancer. Cancer Res.

[171] Duesberg P, Li R, Fabarius A, Hehlmann R (2006). Aneuploidy and cancer: from correlation to causation. Contrib Microbiol.

[172] Sheltzer JM (2011). Aneuploidy drives genomic instability in yeast. Science.

[173] Janssen A, van der Burg M, Szuhai K, Kops GJ, Medema RH (2011). Chromosome segregation errors as a cause of DNA damage and structural chromosome aberrations. Science.

[174] Stephens PJ (2011). Massive genomic rearrangement acquired in a single catastrophic event during cancer development. Cell.

[175] Liu P (2011). Chromosome catastrophes involve replication mechanisms generating complex genomic rearrangements. Cell.

[176] Magrangeas F, Avet-Loiseau H, Munshi NC, Minvielle S (2011). Chromothripsis identifies a rare and aggressive entity among newly diagnosed multiple myeloma patients. Blood.

[177] Crasta K (2012). DNA breaks and chromosome pulverization from errors in mitosis. Nature.

[178] Thompson SL, Compton DA (2011). Chromosome missegregation in human cells arises through specific types of kinetochore-microtubule attachment errors. Proc Natl Acad Sci U S A.

[179] Feinberg AP, Ohlsson R, Henikoff S (2006). The epigenetic progenitor origin of human cancer. Nat Rev Genet.

[180] Esteller M (2008). Epigenetics in cancer. N Engl J Med.

[181] Andriani GA (2016). Whole Chromosome Instability induces senescence and promotes SASP. Sci Rep.

[182] Davoli T (2013). Cumulative haploinsufficiency and triplosensitivity drive aneuploidy patterns and shape the cancer genome. Cell.

[183] Bowers RR (2022). SWAN pathway-network identification of common aneuploidy-based oncogenic drivers. Nucleic Acids Res.

[184] Kawakami M, Liu X, Dmitrovsky E (2019). New cell cycle inhibitors target aneuploidy in cancer therapy. Annu Rev Pharmacol Toxicol.

[185] Tang YC, Williams BR, Siegel JJ, Amon A (2011). Identification of aneuploidy-selective antiproliferation compounds. Cell.

[186] Schukken KM (2020). Altering microtubule dynamics is synergistically toxic with spindle assembly checkpoint inhibition. Life Sci Alliance.

[187] Tang YC (2017). Aneuploid cell survival relies upon sphingolipid homeostasis. Cancer Res.

[188] Janssen A, Kops GJ, Medema RH (2009). Elevating the frequency of chromosome mis-segregation as a strategy to kill tumor cells. Proc Natl Acad Sci U S A.

[189] Kops GJ, Foltz DR, Cleveland DW (2004). Lethality to human cancer cells through massive chromosome loss by inhibition of the mitotic checkpoint. Proc Natl Acad Sci U S A.

[190] Maia AR (2015). Inhibition of the spindle assembly checkpoint kinase TTK enhances the efficacy of docetaxel in a triple-negative breast cancer model. Ann Oncol.

[191] Wengner AM (2016). Novel Mps1 kinase inhibitors with potent antitumor activity. Mol Cancer Ther.

[192] Cohen-Sharir Y (2021). Aneuploidy renders cancer cells vulnerable to mitotic checkpoint inhibition. Nature.

[193] Quinton RJ (2021). Whole-genome doubling confers unique genetic vulnerabilities on tumour cells. Nature.

[194] Marquis C (2021). Chromosomally unstable tumor cells specifically require KIF18A for proliferation. Nat Commun.

[195] Tamayo NA (2022). Targeting the mitotic kinesin KIF18A in chromosomally unstable cancers: hit optimization toward an in vivo chemical probe. J Med Chem.

[196] Rosen LE (2019). Cohesin cleavage by separase is enhanced by a substrate motif distinct from the cleavage site. Nat Commun.

[197] Kumada K (2006). The selective continued linkage of centromeres from mitosis to interphase in the absence of mammalian separase. J Cell Biol.

[198] Wirth KG (2006). Separase: a universal trigger for sister chromatid disjunction but not chromosome cycle progression. J Cell Biol.

[199] Mukherjee M (2011). Separase loss of function cooperates with the loss of p53 in the initiation and progression of T- and B-cell lymphoma, leukemia and aneuploidy in mice. PLoS ONE.

[200] Zhang N (2014). Identification and characterization of separase inhibitors (sepins) for cancer therapy. J Biomol Screen.

